# Transcervical artificial insemination in the brown brocket deer (*Subulo gouazoubira*): a promising method for assisted reproduction in deer

**DOI:** 10.1038/s41598-023-43392-4

**Published:** 2023-10-13

**Authors:** Gabriella Saloni Duarte, David Javier Galindo, Maria Helena Mazzoni Baldini, Jeferson Ferreira da Fonseca, José Mauricio Barbanti Duarte, Maria Emilia Franco Oliveira

**Affiliations:** 1 Núcleo de Pesquisa e Conservação de CervídeosFaculdade de Ciências Agrárias e Veterinárias, Universidade Estadual Paulista, Via de Acesso Prof. Dr. Paulo Donato Castellane, S/N, Jaboticabal, São Paulo, 14884-900 Brazil; 2 Laboratorio de Reproducción Animal, Facultad de Medicina Veterinaria, Universidad Nacional Mayor de San Marcos, Avenida Circunvalación 28,, San Borja, 15021 Lima Peru; 3 Embrapa Caprinos e Ovinos, Estrada Sobral/Groaíras, Km 04, CP 145, Sobral, Ceará 62010-970 Brazil; 4 Departamento de PatologiaReprodução e Saúde ÚnicaFaculdade de Ciências Agrárias e Veterinárias, Universidade Estadual Paulista, Via de Acesso Prof. Dr. Paulo Donato Castellane, S/N, Jaboticabal, São Paulo, 14884-900 Brazil

**Keywords:** Animal biotechnology, Biotechnology, Endocrinology

## Abstract

The present study aimed to test the efficiency of transcervical artificial insemination techniques with cervical immobilization (TCAI-CI) or cervical traction (TCAI-CT), associated or not with the use of oxytocin (OT) as a protocol for cervical dilation, in the brown brocket deer (*Subulo gouazoubira*). The study was carried out in a crossover design using four adult females in two replicates with an interval of 60 days. Estrus was synchronized with oral melengestrol acetate (MGA) associated with estradiol benzoate and sodium cloprostenol. TCAI techniques were performed from 18 to 24 h after estrus onset. All females received either an i.v. application of 50 IU of OT (G-OT, n = 4) or 1 mL of saline solution (G-Control, n = 4) 20 min before the TCAI procedure. The TCAIs were performed using frozen-thawed semen motility 40%, vigor 3, acrosome integrity 87%, membrane integrity of 95% and 13% of total post-thaw defects from the same batch. Behavioral estrus was observed in 100% of the females, in both replicates. It was achieved a 50% (4/8) success of cervical transposition with semen deposition in the uterine. Regarding inseminations, most of them (87.5%) were performed using the TCAI-CT technique, and the overall conception rate was 50%. Cervical transposition times (< 1 min) and TCAI procedures (~ 17 min) were considered satisfactory. Thus, the performance of the TCAI-CI and TCAI-CT techniques was successful, regardless of using OT as a cervical dilation protocol. This procedure is proposed as a method of choice for artificial insemination with greater applicability in different conservation centers, compared to more advanced reproductive biotechniques, and with a favorable impact on the conservation of deer species.

## Introduction

The environmental impact of unsustainable exploitation of natural resources, as well as other threats, has led to biodiversity reduction and, subsequently, has also increased the rate of extinction faster than the rate of speciation^1,2^. In that context, several reproductive biotechnologies appear as part of conservation strategies^3,4^. Among these, germplasm banks are positioned as an essential strategy for *ex-situ* conservation of genetic resources, enabling the preservation of the genetic diversity of wild animals^5,6^.

For animals at risk or threatened with extinction, such as some species of deer, one way to guarantee their existence is to increase their reproductive rates via reproductive biotechnologies. However, the application of these biotechniques to wild species is still minimal, mainly due to the lack of basic knowledge of the reproductive biology of most species, such as deer^7^. In these animals, even in the wild, there is a high degree of structural sperm abnormalities caused by the increase in inbreeding (loss of genetic variability) due to the population decline caused by environmental changes and human interventions^7^. This trend is also observed in captive animals, caused by the stress of the reduced space, change of environment, and constant coexistence with other individuals^8,9^.

An important reproductive biotechnique is artificial insemination (AI), which is widely performed in cervids via laparoscopic access^5,6,8^. However, there are also reports of AI with semen deposition in the vagina without cervical transposition^9^. Laparoscopic methods for AI are reported in several deer species, including *Mazama americana*^9^, *Subulo gouazoubira*^10^ (formerly *Mazama gouazoubira*)^11^, *Dama dama*^12^, *Cervus Eldi thamin*^13^, *Cervus nippon*^13^, *Cervus elaphus*^14^, *Elaphurus davidianus*^15^, *Odocoileus virginianus*^16^, and *Rangifer tarandus*^8^.

Although the efficacy of the intrauterine AI technique by video laparoscopy is satisfactory in deer species, as reported for *S. gouazoubira* with a conception rate of 50% using frozen-thawed semen^10^, this surgical procedure is minimally invasive. It can cause reproductive sequelae, such as adhesions^17^. On the other hand, transcervical AI (TCAI) is a less invasive option and, therefore, more broadly applicable considering animal welfare. However, the biggest obstacle regarding deer TCAI is the anatomy of the cervix, which, similar to sheep, has a tortuous cervical canal with reduced dimensions^6,18^. Despite this, there is one successful report for the cervical canal transposition and semen deposition in the uterine lumen of a *Passalites nemorivagus* (formerly *Mazama nemorivaga*)^19^ female, giving rise to a healthy offspring^20^. Unfortunately, despite this successful report, there have been no reports of other attempts on Neotropical deer.

In sheep, the cervical canal is considered the main obstacle to performing TCAIs^21^. Thus, to simplify cervical transposition before performing TCAI, hormone protocols based on prostaglandins (E_2_ and E_3_)^22^ and a combination of oxytocin (OT) and estradiol^23,24^ have been tested in sheep. In parallel, the development of specific instruments and techniques for AI in sheep and goats have also facilitated obtaining greater efficiency in procedures performed through the transcervical route. In goats, the cervical immobilization technique using the EMBRAPA forceps is effective in ensuring a high rate of cervical transposition during TCAI procedures and for transcervical embryo collection (95% and 100%, respectively)^25^.

On the other hand, due to the more significant tortuosity of the ovine cervical canal when compared to goats, it was necessary to perform the cervical clamping/traction technique using Pozzi forceps. This was preceded by the cervical dilation protocol composed of a combination of estradiol benzoate, d-cloprostenol, and OT, obtaining high cervical transposition rates (80–100%) during transcervical embryo collection procedures^26–29^. Equivalent cervical transposition success in transcervical embryo collection was achieved by reducing the estradiol benzoate dose (1.0 vs. 0.5 mg) or eliminating its use in sheep with synchronized estrus, maintaining the association with d-cloprostenol and OT^30^.

Among the Neotropical deer species, brown brocket deer (*S. gouazoubira)* is one of the most abundant species, both in nature and in captivity. It also has a wide distribution in South America and is classified as “Least Concern” by the IUCN^31^. Thus, it has been considered an excellent experimental model for threatened deer species (10 of the 17 neotropical deer species)^32^. The present study aimed to test the efficiency of two TCAI techniques, either with cervical immobilization or cervical traction, associated or not with the use of OT as a protocol for cervical dilation in *S. gouazoubira* females.

## Materials and methods

### General experimental condition

The Ethics Committee on Animal Use (CEUA) of the School of Agricultural and Veterinarian Sciences from São Paulo State University (Unesp), Jaboticabal, São Paulo, Brazil, approved all animal procedures (protocol number 015312/19) and all methods were performed in accordance with the relevant guidelines and regulations by including a statement in the methods section to this study. We declare that the manuscript complies with the ARRIVE guidelines. This is to the ethical principles adopted by the National Council for the Control of Animal Experimentation (CONCEA). The study was carried out in a crossover design between October 2020 and January 2021, with intervals of 60 days between the end of the first treatment and the beginning of the second so that all females belonged to both experimental groups. Four brown brocket hinds (*S. gouazoubira*) were kept in individual pens (12 m^2^) with olfactory and sound contact with conspecific females and males, exposed to natural photoperiod fluctuation at the Deer Research and Conservation Center (NUPECCE). Animals were fed with 0.5 kg of pelleted ration diet for horses (Equitech®—Presence®—Paulinia, São Paulo, Brazil) provided once daily in the morning. Approximately 1 kg/deer/day of perennial soybean (*Neonotonia wightii*), ramie (*Boehmeria nivea*), or blackberry branches (*Morus alba*), provided according to their availability in the field. Water was offered ad libitum.

### Estrus synchronization protocol

**Figure 1:**
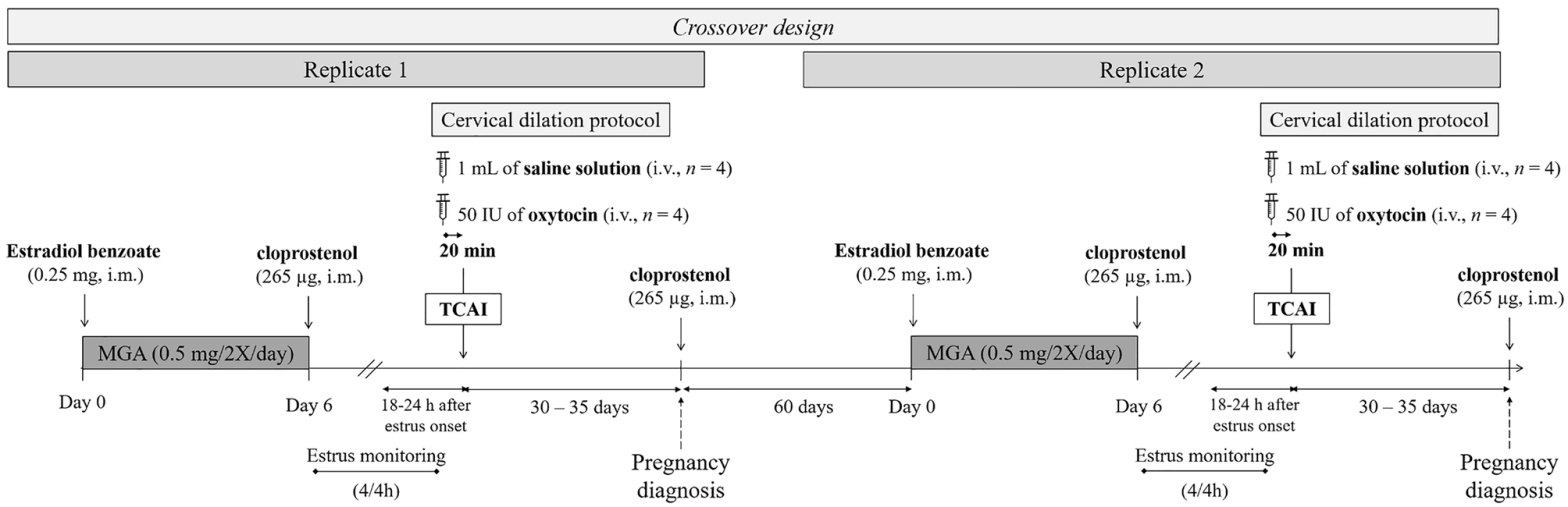
The usual schedule of the experimental design applied for testing the efficiency of transcervical artificial insemination techniques, with cervical immobilization or cervical traction, in *Subulo gouazoubira* females submitted or not to a cervical dilatation protocol with oxytocin. MGA, melengestrol acetate; TCAI, transcervical artificial insemination.

Estrus synchronization was based on the hormone protocol described by Tanaka et al. (2020)^33^. All animals received two daily doses of 0.5 mg of melengestrol acetate (MGA, Premix ®; Pfizer, São Paulo, Brazil) for seven days, considering Day 0 as the start of the estrus synchronization protocol. MGA doses were mixed with mashed banana, an edible fruit for deer. Also, on Day 0, females received an i.m. injection of 0.25 mg of estradiol benzoate (Sincrodiol®; Ourofino Saúde Animal Ltda., Cravinhos, Brazil). Concomitantly with the last dose of MGA, an i.m. injection of 265 μg of cloprostenol sodium (Ciosin®, MSD., São Paulo, Brazil) was applied (Fig. 1). Between Days 7 and 10, females were subjected to estrus detection every four hours, with the aid of a fertile male of the same species. One animal, which was more docile, was evaluated using lordosis response to handlers and vaginal mucus evaluation^6^. The estrus rate ([%, number of females in estrus/number of females synchronized] × 100) and the interval from treatment to estrus onset (behavioral estrus detection after administration of cloprostenol sodium, in hours) were calculated.

### Cervical dilation protocol and transcervical artificial insemination (TCAI)

Artificial inseminations were performed between 18 to 24 h after the estrus detection, considering insemination in the final third of the estrus according to the estrus duration in the species^10^. Twenty minutes before the TCAI procedure, the females received an intravenous administration of 50 IU of OT (Ocitocina forte UCB®, UCB, Jaboticabal, Brazil) (G-OT, *n* = 4) or 1 mL of saline solution (G-Control, *n* = 4). These drugs were administered in a double-blind trial so that the professional inseminator would not know to which group the females belonged. The OT dose was determined based on Dias et al. (2020)^30^.

Females were subjected to chemical restraint by i.m. of 7 mg/kg of ketamine hydrochloride (Cetamin®, Syntec, Santana do Parnaíba, Brazil) and 1 mg/kg of xylazine hydrochloride (Xilazin®, Syntec, Santana do Parnaíba, Brazil). For analgesia, a slow infusion of 2.5 µg/kg of fentanyl citrate (Fentanest®, São Paulo, Brazil) and the application of 0.4 mg/kg of dipyrone/hyoscine mixture (Buscofin Composto®, Agener União, Embu Guaçu, Brazil) were administered during and after the procedure, respectively.

Females were positioned on a procedure table in sternal recumbency, maintaining the pelvis position with the pelvic limbs flexed. Attempts on cervical transposition were performed within TCAI procedures through cervical immobilization (TCAI-CI) and cervical traction (TCAI-CT). The two procedures were conducted sequentially on all females in both treatments. When the uterus was reached, semen deposition was performed.

**Figure 2:**
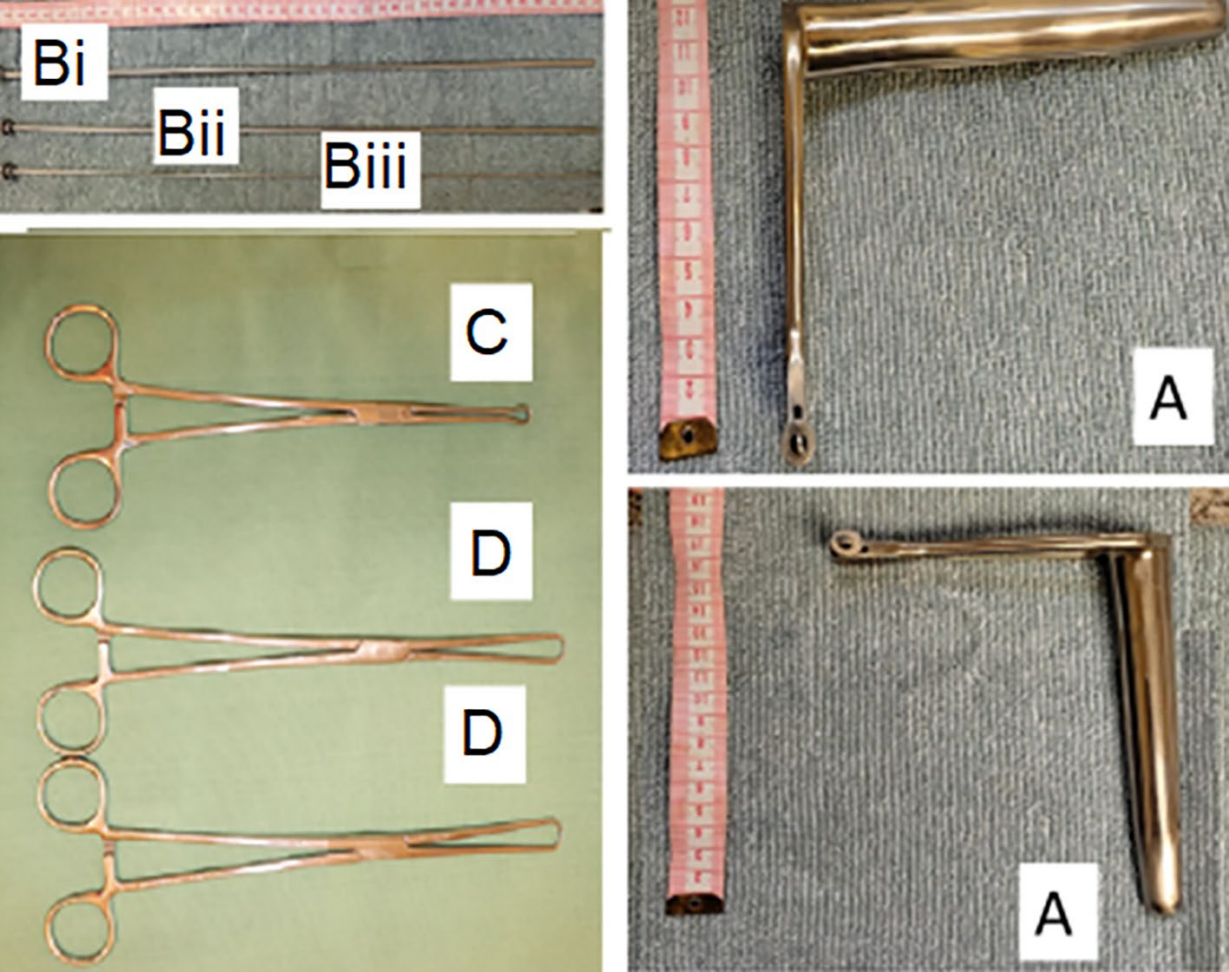
Instruments, a speculum (**A**) and semen applicator (**Bi**) with expander mandrel (**Bii**) and the applicator mandrel (**Biii**), developed for *Subulo gouazoubira* females, (**C**) Embrapa forceps, Pozzi forceps (**D**).

**Figure 3:**
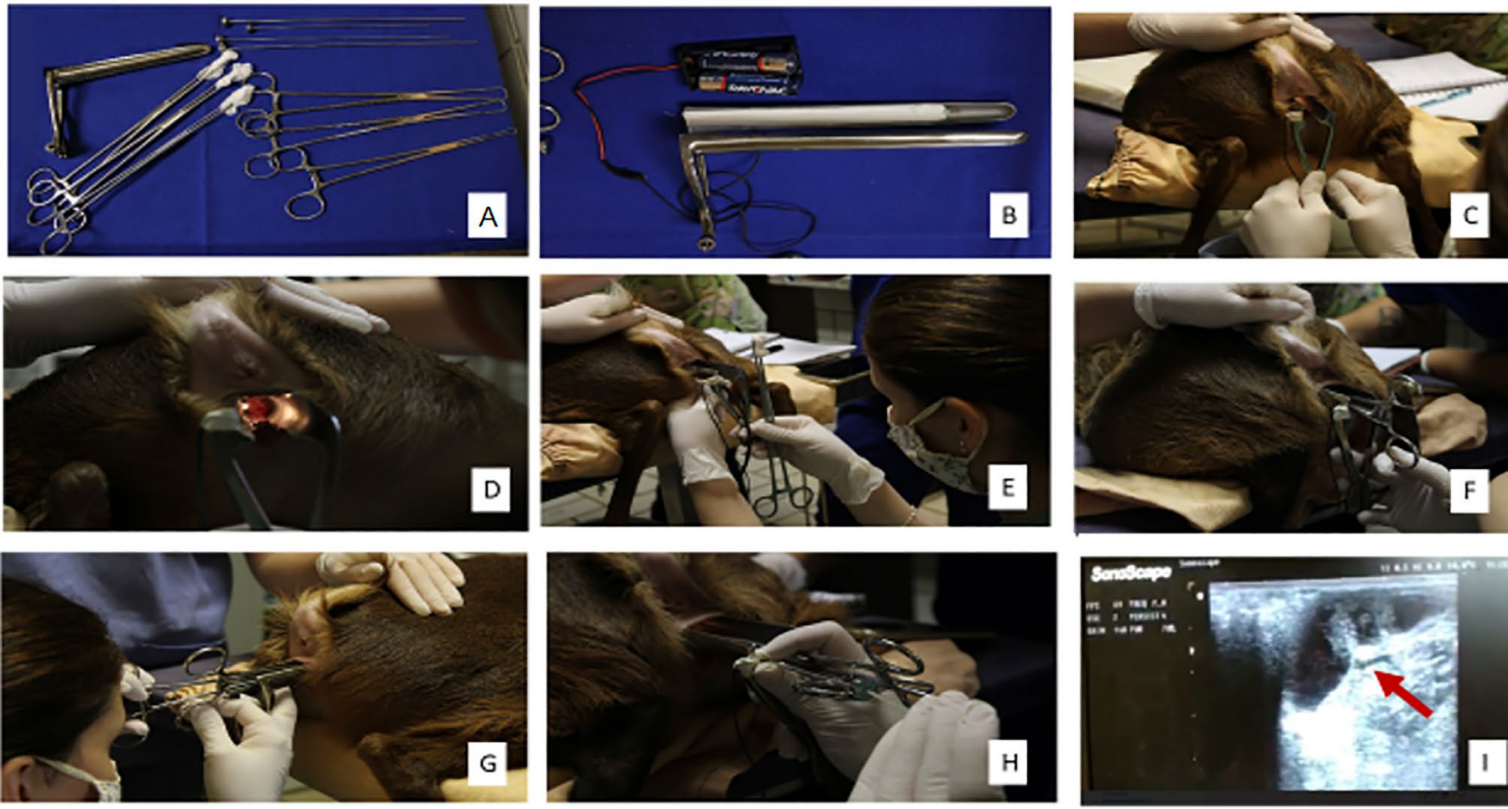
Sequential images of the artificial insemination procedures performed on *Subulo gouazoubira* females. Surgical instruments preparation (**A**); vaginal speculum with a light source (**B**); introduction of the vaginal speculum (**C**); cervical ostium visualization (**D**); local anesthesia with a cotton swab with 5 ml of 2% lidocaine in the bottom of the vaginal fornix (**E**); TCAI-CI with fixed Embrapa forceps (**F**); TCAI-CT with fixed Pozzi forceps (**G**); semen applicator (**H**); and pregnancy diagnosis 30 days after TCAI, showing an image of the embryonic cyst (arrow) in the uterus (**I**).

The techniques presented followed the previously described for TCAI-CI in goats^25^ and TCAI-CT in sheep^27^. After surgical table organization (Fig. 3A), external cleaning and antisepsis of the perineal and vulvar region, a sterile gauze soaked with 5 ml of 2% lidocaine (2% Lidocaine Chloridate®, Bravet, Rio de Janeiro, Brazil) (Fig. 3E) was gently placed at the bottom of the vaginal fornix for 2 min. Then, a vaginal speculum manufactured for the species (17 cm long and with light source attached; (Fig. 2A) (Fig. 3B), with non-spermicidal intimate lubricant (K-Y®, Semina, São Paulo, Brazil), was slowly inserted (Fig. 3C,D). The vagina length (cm) was determined by marking the semen applicator with a marker (Fig. 2Bi,Bii). The cervical presentation was classified according to Kershaw et al. (2005)^21^ and the mucus was evaluated for presence, color, appearance (crystalline, crystalline/striated, striated, striated/caseous, and caseous)^25^ and quantity (1—tiny, 3—abundant). The grade projection of the cervix into the speculum was rated from 1 to 3 (Fig. 3D) (1—poor projection and 3—good projection).

In the TCAI-CI technique (Fig. 3F), a costume-made 25 cm long Embrapa forceps (Embrapa forceps for CI and AI in small ruminants, Brasília, Brazil; Fig. 2C)^27^ was inserted inside or under the cervical os or just pinched ventrally, according to the anatomy of the cervical os of each animal (Fig. 3F). In the TCAI-CT (Fig. 3G), one or two 26 cm long Pozzi forceps were fixed laterally to the cervical os (Fig. 2D) (Fig. 3G), according to the grade of vaginal distension by the speculum. The CT was carried out until reaching every animal's anatomical limit. The difficulty for clamping and traction was classified from 1 (easy immobilization) to 3 (rigid immobilization). Clamping repetitions were made whenever clamping got loose or the cervix positioning did not allow performing the following technique steps. All recurrences were recorded. The variables related to clamping were determined for the TCAI-CI and TCAI-CT techniques.

Cervical transposition attempts were performed with a semen applicator manufactured for the species (17 cm long and with two mandrel options, a Hegar uterine dilator, and a semen applicator) (Fig. 2Bi,Bii,Biii) (Fig. 3H). During the cervical transposition attempt, the Hegar uterine dilator (Fig. 2Bi,Bii) (Fig. 3H) was kept in the applicator (Fig. 2Biii). The length of the transposed cervix (cm), the number of cervical rings transposed, and the grade of tortuosity of the cervical canal (1—straight to 3—great tortuosity) were recorded. The degree of difficulty of cervical transposition (1—easy to 3—impossible to reach the uterus), the percentage of females in which cervical transposition was successful (complete transposition), the time for achieving cervical transposition (in minutes, from the semen applicator, settled up in the cervical os until reaching the uterus, or when exceeding 2 min off transposition attempts), and duration of the AI procedure (in minutes, since the introduction of the speculum until semen deposition or exceeding the transposition time) were determined for the TCAI-CI and TCAI-CT techniques. The percentage of females inseminated according to the place of semen deposition (vaginal, superficial cervical, deep cervical, and uterine), percentage of occurrence of semen reflux, and conception rate was also calculated.

Insemination was performed with a 0.25 mL straw of frozen/thawed semen from the same male and batch, with a total concentration of 50 × 10^6^ spermatozoa/paw. Semen collection was performed by electroejaculation, from 250 to 750 mA in a stimulation cycle of 3 s of application and 3 s of rest, totaling 10 stimuli. The diluents for semen cryopreservation were carried out using the recipe of tris-citric acid with 2.25% or 20% of egg yolk and each sample was thawed in a water bath at 37 °C for 20 s and evaluated according to the parameters and demonstrated: motility 40%, vigor 3, acrosome integrity 87%, membrane integrity 95% and 13% total defects after thawing. After insemination and removal of instruments, the female had her pelvic region elevated for 2 min and received clitoral massage.

### Pregnancy diagnosis

Between 30 to 35 days after AI, all females underwent pregnancy diagnosis by transrectal ultrasonography with a 10-MHz linear transducer (Sonoscape A5v® Domed, Valinhos, São Paulo, Brazil) (Fig. 3I), and the conception rate was calculated. Pregnant females received an i.m. 265 µg of cloprostenol sodium for the induction of luteolysis and embryonic loss to allow the realization of the second replica with the same animals.

### Statistical analysis

All results are expressed as mean ± standard deviation or percentage. Due to the low number of available individuals, a common issue within wild animal research, only descriptive data analysis was performed.

## Results

All females in the two replicates showed estrus behavior 44.0 ± 22.0 h after the end of the estrus synchronization protocol (cloprostenol administration). The mean vagina length was 13.0 ± 1.2 cm, and 75% (3/4) of the females showed a papillary cervix os, while 25% (1/4) showed a duckbill cervix os. The presence of translucent mucus was observed 18–24 h after estrus detection in 100% (4/4, G-control) and 75% (3/4, G-OT) of the females, with a 100% crystalline/striated appearance in females of the G-Control and 50% crystalline and 50% crystalline/striated for females of the G-OT. The mean amount of mucus (1–3) was 1.2 ± 1.2.

**Figure 4:**
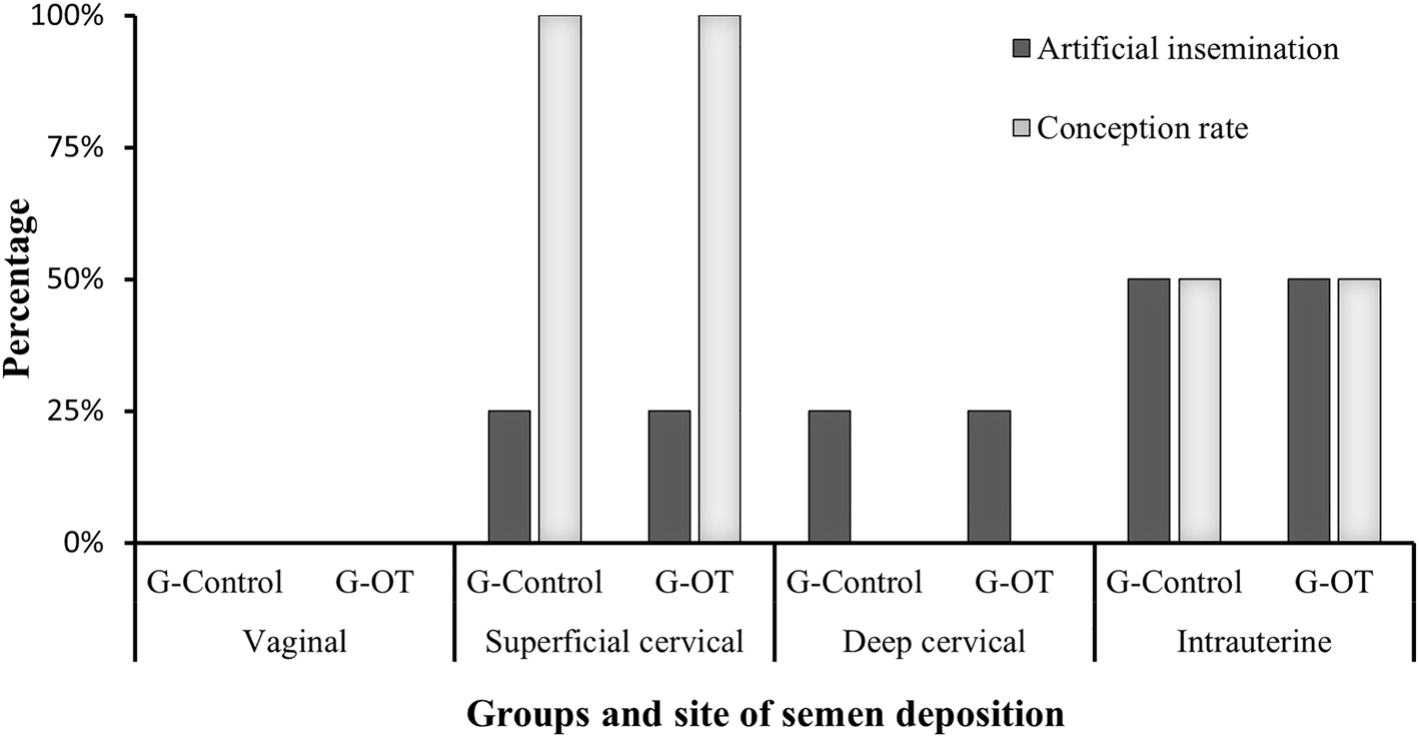
Females inseminated by the transcervical route (%) and conception rate (%) according to the site of semen deposition, submitted or not to a cervical dilation protocol with oxytocin (G-Control, 1 mL de saline solution; G-OT, 50 IU of Oxytocin). Both were administered intravenously 20 min before the AI procedure.

The results of the efficiency variables of the cervical transposition and TCAI procedures according to the G-Control and G-OT groups are shown in Table 1. The degree of cervix projection on the speculum was considered intermediate (1.7 ± 0.3, score 1–3) and the degree of difficulty of clamping in the cervical traction technique was easer (1.1 ± 0.1, score 1–3). The tortuosity of the cervical canal was registered as maximum (3.0 ± 0.0, score 1–3) and the degrees of difficulty of cervical transposition in both TCAI techniques was high (2.6 ± 0.5, score 1–3). Success of cervical transposition were reached in 12.8% and in 37.5% of females using the cervical immobilization technique and the cervical traction technique, respectively. Times for TCAI were 9.2 ± 0.2 min, using cervical immobilization procedure, and 16.9 ± 0.6 min, using cervical traction procedure. The total conception rate was 50%. In Fig. 4 are showed the percentages of inseminated females conceived according to the semen deposition site. Females that became pregnant had superficial cervical or intrauterine semen deposition.

**Table 1:** Results (mean ± standard deviation) of the efficiency of transcervical artificial insemination techniques^a^, by cervical immobilization or cervical traction, in *Subulo gouazoubira* females submitted or not to a protocol of cervical dilatation with oxytocin (G-Control, 1 mL de saline solution; G-OT, 50 IU of Oxytocin. Both were intravenous administered 20 min before the AI procedure).

Final points	G-Control	G-OT	Total
Estrus response (%)	100%	100%	100%
Time to estrus (hours after cloprostenol administration)	16 ± 24	16 ± 24	16 ± 24
Degree of cervix projection on the speculum (1–3)*	2.0 ± 1.0	1.5 ± 0.6	1.7 ± 0.3
Degree of difficulty of clamping in the cervical traction technique (1–3)*	1.0 ± 0.0	1.2 ± 0.5	1.1 ± 0.1
Number of repetitions of clamping in the cervical immobilization technique	5.0 ± 3.6	5.0 ± 2.4	5.0 ± 0.0
Number of repetitions of clamping in the cervical traction technique	3.3 ± 2.3	4.0 ± 1.4	3.6 ± 0.5
Length of the transposed cervix (cm)	5.9 ± 1.7	4.0 ± 2.6	4.9 ± 1.3
Number of transposed cervical ring	3.5 ± 1.7	1.5 ± 1.0	2.5 ± 1.4
Degree of tortuosity of the cervical canal (1–3)*	3.0 ± 1.0	3.0 ± 1.0	3.0 ± 0.0
Degree of difficulty of cervical transposition in the cervical immobilization technique (1–3)*	2.2 ± 0.9	3.0 ± 0.0	2.6 ± 0.5
Degree of difficulty of cervical transposition in the cervical traction technique (1–3)*	2.2 ± 0.9	3.0 ± 0.0	2.6 ± 0.5
Success (%) of cervical transposition in the cervical immobilization technique	25% (1/4)	0% (0/4)	12.5% (1/8)
Success (%) of cervical transposition in the cervical traction technique	25% (1/4)	50% (2/4)	37.5% (3/8)
Time for cervical transposition in the cervical immobilization technique (min)	0.6 ± 0.1	1.1 ± 0.5	0.8 ± 0.3
Time for cervical transposition in the cervical traction technique (min)	0.5 ± 0.4	0.2 ± 0.2	0.3 ± 0.2
Time for TCAI—cervical immobilization procedure (min)	9.4 ± 4.0	9.1 ± 2.5	9.2 ± 0.2
Time for TCAI—cervical traction procedure (min)	17.4 ± 2.5	16.5 ± 4.2	16.9 ± 0.6
Occurrence of semen reflux (%)	50% (2/4)	0% (0/4)	25% (2/8)
Conception rate (%)	50% (2/4)	50% (2/4)	50% (4/8)
Artificial insemination procedures using EMBRAPA forceps (%)	25% (1/4)	0% (0/4)	12.5% (1/8)
Artificial insemination procedures using Pozzi forceps (%)	75% (3/4)	100% (4/4)	87.5% (7/8)

^a^TCAI was performed 18 to 24 h after the detection of behavioral estrus. All females were submitted to a Melengestrol Acetate-based estrus synchronization protocol. * 1: easy; 3: hard. ( ): animal ratio.

## Discussion

This is the first TCAI study in females of *S. gouazoubira*, achieving success on cervical transposition with semen deposition in the uterine body in 50% (4/8) of the procedures. Most inseminations (87.5%) were performed using the TCAI-CT technique, and the overall conception rate was 50%. The performance index of the TCAI-CI and TCAI-CT techniques points out the success of the non-surgical artificial insemination procedures in *S. gouazoubira* females, regardless of the use of oxytocin as a cervical dilatation protocol.

Due to a high stress stimulus when handling the animal to administer the drugs^8,34,35^, which could alter their reproductive physiological patterns^36^, oral synthetic progestagens, such as melengestrol acetate (MGA), are an efficient option for estrus synchronization in deer species^33,37^ and domestic ruminants^38–41^. In the present study, 100% of females showed behavioral estrus approximately 44 h after the end of the protocol. Similar success (100%) was previously reported for *S. gouazoubira* using the same oral progesterone^33^. Thus, the MGA-based estrus synchronization protocol is indicated for its high efficiency and for improving the welfare of the managed females, reducing stress sources, and offering more excellent safety to animals and technicians^38–41^. In deer, an epidural block is problematic and stressful during the post-procedure period, when the animal may recover from sedation without return pelvic limb movement. Thus, in addition to the chemical restraint protocol traditionally used by NUPECCE^20^, the association of a slow infusion of fentanyl citrate (2.5 µg/kg) was performed to mitigate the signs of discomfort during the intraoperative period. After the procedure, the females received dipyrone/hyoscine (0.4 mg/kg)^27^ to prevent post-procedural pain sensitivity, as done in sheep.

The TCAI techniques of the present study have great potential for use in other deer species in different conservation centers (Zoos, Breeding Institutions, and Scientific Breeding Institutions) due to their greater practicality compared to other techniques, such as laparoscopy or laparotomy. Thus, they appear as a practical and replicable alternative for the genetic management of a great variety of captive populations, which are necessary genetic resources for the conservation of free-living populations and so the species^3,20^. Moreover, the chosen TCAI techniques showed satisfactory results in brow brocket females. However, it was necessary to develop new instruments considering the anatomical particularities of the species. Thus, a speculum with a smaller diameter and greater length and a longer semen applicator, based on TCAI-CI for goats^25^ and TCAI-CT for sheep^27^, were manufactured.

Moreover, using the Embrapa forceps ensured the successful application of the two TCAI techniques (TCAI-CI, and TCAI-CT). Regarding the TCAI-CI, the immobilization was performed only with the Embrapa forceps. While in the TCAI-CT, Embrapa forceps were used before the Pozzi forceps were fixed, which enabled traction movements in most procedures, being removed soon after.

Some anatomical characteristics, such as vagina length and presentation of the cervical *os*, have not been previously described in this species. The vagina length determined for *S. gouazoubira* in the present study was ~ 13 cm, longer than that reported for sheep (4–7 cm^42^). The greater depth of the vagina in the brown brocket animals required the development of specific instruments, such as a vaginal speculum and a semen applicator. The observed services had conformation of papilla and duckbill type and did not limit the performance of cervical clamping procedures, either using the Embrapa forceps or the Pozzi forceps. The length of the transposed cervix (~ 5 cm) might be used as a cervical length indicator for *S. gouazoubira* females. Nevertheless, the number of cervical rings counted (mean of ~ 2.5) seems to have been underestimated, considering the high degree of difficulty for the cervical transposition.

Cervical mucus was observed in most females (87.5%), confirming that they were in estrus. The mucus appearance is a physiological characteristic evaluated to determine the time of ovulation in small domestic ruminants, this being more accurate in goats^25^ than in sheep^43^. Compared to data from domestic species, the mucus observed in the present study (crystalline and crystalline/striated) is related to initial moments of estrus, preceding ovulation by ~ 40 hours^25^, and may be an indicator of estrus synchrony among them. Regarding Neotropical deer species, there is a lack of data on the moment ovulation occurs in response to estrus synchronization protocols. However, most AI performed at NUPECCE were between 18 to 24 h after the estrus onset, which is also considered the ideal time for AI after behavioral estrus detection in sheep/goat artificial insemination is performed 18 to 24 h after CIDR removal.

The main anatomical limitation found for performing AI procedures in brown brocket animals, aside from the longer vaginal length, was the low grade of vaginal distension. Despite some projection of the cervix towards the interior of the speculum, the outer end of the cervix did not reach the vulvar rim. Therefore, the clamping was carried out while still inside the vaginal canal. These characteristics highlighted the need for specific instruments for the species and well-trained technicians to perform the procedures. According to Fonseca et al. (2011)^44^, obtaining an excellent cervical projection into the speculum is essential for performing transcervical inseminations. It can be facilitated by adjusting the speculum size for each female. Among the females included in this study, no variation in the diameter of the vagina would indicate the use of different speculum sizes. The set of anatomical characteristics that restricted the space for manipulation of the cervix was a challenge for the performance of TCAI in *S. gouazoubira*, demonstrated by the need to repeat the clamping between 3 and 5 times per procedure.

The cervical canal tortuosity in brown brocket females was classified at the maximum level, indicating a high degree of tortuosity. In sheep, the cervix is described as a tubular, narrow, long, and tortuous organ^45^. Among brown brocket females, different patterns of topographic distribution of the cervical os can be found^27^, leading to greater or lower ease of cervical transposition. The CT can lead to greater alignment of the cervical canal compared to a technique that uses only CI^25^. In the present study, the exclusive use of OT before the procedures suggests an improvement in the percentage of cervical transposition only in the CT technique. Failure of cervical transposition by the CI technique in the G-OT may result from not only the lack of OT effect on dilating the cervical canal but also the lack of cervical alignment, thus making it difficult to perceive the dilation of the cervical rings. The cervical transposition times (< 1 min) and TCAI procedures (~ 17 min, from speculum placement to semen deposition) are satisfactory and encouraging for the dissemination of the use of TCAI techniques performed in the present study in *S. gouazoubira* females, as well as in other deer species. In sheep, 83.5% of intrauterine semen deposition was recorded in a mean time of 32 s for cervical transposition ^46^.

The overall conception rate of females was 50%, regardless of whether they had previously received OT. Similar pregnancy success rates were previously reported after laparoscopic artificial insemination in the same deer species (50%)^10^ and for females of *Odocoileus virginianus* (45%)^47^ and *Dama dama* (56%)^48^. Oxytocin did not interfere with the success rates of TCAI techniques, except for the apparent reduction in the occurrence of semen reflux. The occurrence of reflux is possibly influenced by the location of semen deposition (more abundant when the insemination is more superficial), which may also decrease the number of sperm that reach the fertilization site^49^.

In the present study, conception occurred in 100% of females where artificial insemination was superficial cervical and in 50% of females with intrauterine semen deposition (Fig. 4). In addition to the satisfactory results on cervical transposition and intrauterine semen deposition, the duration of the procedure and conception rate support the indication of these techniques in *S. gouazoubira* females, regardless of the use of oxytocin as a promoter of cervical dilatation. The use of minimally invasive procedures, such as those applied in the present study, is widely demanded today, mainly in societies concerned with animal welfare issues^50^. The new techniques described have a lower cost and shorter procedure time if compared with videolaparoscopy technique. Furthermore, the TCAI techniques dispensing with a deep anesthetic plane, enabling the application of the technique in the field, without causing post-procedure adhesions and future loss of fertility. Thus, we believe that the TCAI techniques reported for the first time in *S. gouazoubira* can improve the perspective of reproductive biotechnologies in this species of deer and consequently contribute to the preservation of endangered species such as *Mazama nana* and *Mazama jucunda.*

## Conclusion

A minimally invasive estrus synchronization protocol for *Subulo gouazoubira* females was successfully performed in the present study. Subsequently, artificial insemination technique with cervical immobilization or traction were successfully applied to *Subulo gouazoubira* females, standing out as promising techniques for the conservation and genetic management of threatened deer species. Most inseminations were performed using the TCAI-CT method (87.5%), pointing this out as an encouraging and potentially applicable method for future embryo collection in deer. The exclusive use of oxytocin as a promoter of cervical dilation does not seem to have increased the success rates of the technique, suggesting that the use of dilators during estrus is not necessary, which may be due to the estrus phase where there is already an increase in estrogen and consequent cervical dilation. All the procedures presented in this study are designed to enable their replication, independent of the reality of the conservation center where they are carried out. This is due to their low cost and low demand for extremely sophisticated materials. However, a key point will be having highly trained personnel to carry out these TCAI techniques (Supplementary Information 1).

In addition, the technique have a lower cost, more superficial anesthetic plane, shorter procedure time, do not present a decrease in fertility and have a more viable applicability in free living animals, when compared to previous videolaparoscopy technique.

### Supplementary Information

Supplementary Information.

## Data Availability

Datasets used and/or analyzed during the current study made available by the author correspond upon reasonable request.
